# The influence of hydrogen bonding on the structure of organic–inorganic hybrid catalysts and its application in the solvent-free epoxidation of α-olefins†

**DOI:** 10.1039/d4ra01399a

**Published:** 2024-04-22

**Authors:** Hong-Bin Ju, Li-Zhi Zhang, De-Bao Li, Tao Geng, Ya-Jie Jiang, Ya-Kui Wang

**Affiliations:** a Institute of Coal Chemistry, Chinese Academy of Sciences Taiyuan 030001 Shanxi China dbli@sxicc.ac.cn; b China Research Institute of Daily Chemistry Co., Ltd Taiyuan 030001 Shanxi China anhuayanjiushi@163.com; c China Research Institute of Daily Chemical Industry Taiyuan 030001 Shanxi China; d University of Chinese Academy of Sciences Beijing 100049 China; e Shanxi Key Laboratory of Functional Surfactants Taiyuan 030001 Shanxi China

## Abstract

In this study, two types of catalysts were prepared by the combination of gemini quaternary ammonium salt with two distinct species of phosphotungstic acid. Catalysts prepared by the Wells–Dawson type of phosphotungstic acid and Keggin-type phosphotungstic acid both exhibited dual-phase catalytic behavior, demonstrating both heterogeneous and homogeneous catalytic activities. In comparison to the catalyst prepared by the Keggin-type phosphotungstic acid, due to the higher size of Wells–Dawson type of phosphotungstic acid, hydrogen bonding could not effectively affect the catalyst prepared by H_6_P_2_W_18_O_62_. Subsequently, the influential factors on the catalytic reaction were investigated. Through the utilization of techniques such as XPS, FT-IR, Raman spectra and other characterization methods, two distinct structure and reaction mechanisms for these catalysts were elucidated under the influence of hydrogen bonding.

## Abstract 2

Different reaction routes with the influence of hydrogen bonding and without hydrogen bonding.

## Graphical abstract

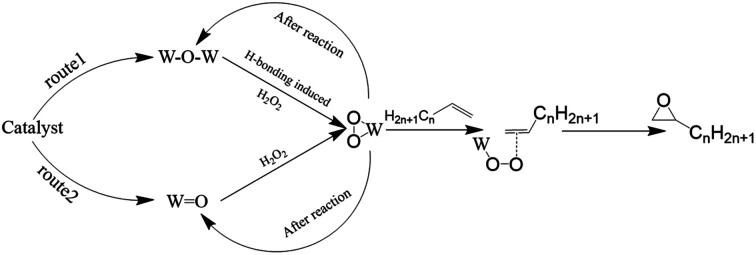

## 1. Introduction

Epoxides represent crucial raw materials for the synthesis of fine chemicals, finding extensive applications in nanomaterials, surfactants, food additives, and cosmetics.^1,2^ Industrially, olefin epoxidation has consistently remained the primary method for producing epoxides. The 1,2-epoxy alkanes synthesized by α-olefins serve as specific intermediates for synthesizing high-value chemicals,^3,4^ presenting a distinct application potential, particularly in the realm of surfactants.^5,6^

In the context of olefin epoxidation, catalysts have played a pivotal role.^7,8^ Broadly, these catalysts can be categorized as either metal-based or inorganic catalysts.^9,10^ Among metal-based catalysts, transition metal-based catalysts have garnered extensive attention owing to their efficient atomic utilization of oxidants.^11^ Typically, these catalysts are prepared into metal–organic frameworks,^6^ polymers,^12,13^ phase-transfer catalysts (PTCs),^14^ and microemulsions^15^ to enhance their catalytic efficiency. The majority of these catalysts are heterogeneous in nature, they may pose certain risks due to the rigorous reaction conditions required for achieving high yields.^16^ Furthermore, the limited solubility of certain types of homogeneous catalysts under catalytic conditions, along with challenges in separation processes, present significant hurdles for researchers.^17^

As a kind of widely used phase-transfer catalysts (PTCs), Ishii–Venturello catalysts^18^ have gained widespread use in epoxidation, owing to their mild catalytic conditions and recyclable characteristics.^19^ Prepared by heteropoly acid and ammonium salt,^20^ these catalysts offer a blend of advantages associated with both heterogeneous and homogeneous catalysts. However, the addition of solvents in the catalytic process for catalyst protection contradicts the principles of green chemistry.^21,22^ Catalysts developed for olefin epoxidation under solvent-free conditions were generally only used to oxidize cycloolefins^23^ or require more stringent reaction conditions.^24^ Consequently, the present research goals lie in enabling catalysts to function without the addition of solvents.

PTCs prepared by polyoxometalates and Gemini quaternary ammonium salts exhibited an enhanced ability to penetrate the oil–water interface to the oil phase under solvent-free conditions. Not only did they demonstrate exceptional catalytic performance, but also facilitated easier recovery from the reaction system.^25,26^ Among these catalysts, the utilization of Keggin-type polyoxometalates (POMs) was prevalent due to their outstanding oxidation catalytic performance.^27,28^ In theory, by the formation of hydrogen bonds in POM, on the one hand, suggesting strong host–guest interactions in two substances could be enhanced,^29^ on the other hand, such strong interactions tightened interface contact and accelerated interface electron transfer of catalyst,^30^ making the catalytic performance more effective. Wells–Dawson-type phosphotungstic acid, characterized by its nanoscale dimensions of approximately 1 nm, can be viewed as an enhanced Keggin-type cluster, offering a greater number of organic ligands.^31,32^ Additionally, it provides more redox active sites. Despite these advantages, this species has not been as extensively employed in epoxidation reactions.^33,34^

In this study, two different types of organic–inorganic composite catalyst was prepared, incorporating Wells–Dawson-type phosphotungstic acid and Keggin-type phosphotungstic acid in combination with a gemini quaternary ammonium salt. Compared with our previous study,^35^ due to the addition of hydroxyl groups in the quaternary ammonium salt, the new catalyst prepared by Keggin-type phosphotungstic acid showed distinct microstructure and reaction mechanism. Subsequently, a comparative analysis was performed between catalysts prepared by Keggin type and Wells–Dawson type phosphotungstic acid, leading to the deduction of distinct reaction mechanisms underlying these catalysts.

## 2. Material and methods

### 2.1 Materials

1,3-Dichloro-2-propanol, propanol, sodium carbonate, 1-dodecene, 1-octene, 1-tetradecene, 1-hexadecene, hydrogen peroxide, phosphotungstic acid, phosphoric acid ethyl alcohol, and methyl alcohol were purchased from Aladdin. Diethyl ether, dodecyl-tetradecyl-dimethylamine, and sodium tungstate were purchased from Wilmar. The above reagents were analytically pure samples. The standard samples used for gas chromatography (1-dodecene, 1-octene, dodecane epoxide and epoxyoctane) were chromatographically pure and purchased from Aladdin.

### 2.2 Synthesis of surfactant DDC

**Scheme 1:**
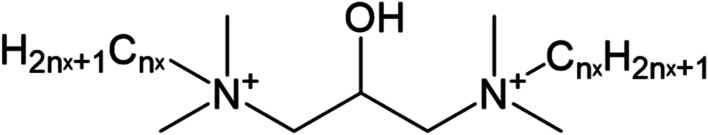
Structure of surfactant DDC, *n*_1_ = 12, *n*_2_ = 14.

The chemical structure of *N*1,*N*3-didodecyl,tetradecyl-2-hydroxy-*N*1,*N*1,*N*3,*N*3-tetramethyl-1,3-propane-diaminium chloride (DDC) was depicted in Scheme 1, which was a type of mixture, with the 3 : 1 molar ratio of dodecylamine to tetradecylamine. The synthesis was conducted under oxygen-free conditions. Specifically, 2 mol of 1,3-dichloro-2-propanol were added into a reactor containing 0.4 mol of dodecyl, tetradecyl-dimethylamine in 70 mL of propanol, with a total mass of 0.3 wt% sodium carbonate acting as the catalyst. The reaction mixture was then refluxed at 60 °C for a period of 4–6 hours. After the reaction, a white precipitate was obtained through vacuum distillation for solvent removal, followed by 3 times recrystallization using a solvent mixture of ethyl acetate and ethanol (20 : 1).

### 2.3 Synthesis of Wells–Dawson type phosphotungstic acid

Wells–Dawson phosphotungstic acid was synthesized by the documented method:^36^ 50 g NaWO_4_·2H_2_O was dissolved in 60 mL distilled water, then poured 35.0 mL of concentrated phosphoric acid. The mixture was heated for 20 minutes until it turned milky white, then further heated to 120 °C for 8 hours. After the reaction, the mixture was cooled down to room temperature, and a specific quantity of concentrated hydrochloric acid was added to acidify the mixture. Diethyl ether was added to extract the phosphotungstic acid. Upon phase separation, the lower diethyl ether phase, consisted with diethyl ether, water and phosphotungstic acid were selected, and diethyl ether was evaporated under 40 °C conditions. The resulting residue was subjected to drying until a constant weight was achieved, yielding the final light yellow solid H_6_P_2_W_18_O_62_·14H_2_O.

### 2.4 Catalyst preparation

1 mmol H_6_P_2_W_18_O_62_·14H_2_O was dissolved in the 20 mL H_2_O_2_ (30wt%), and solution of 3 mmol of the surfactant DDC in 20 mL of anhydrous ethanol was also prepared. The anhydrous ethanol–DDC mixture was then added to the H_6_P_2_W_18_O_62_–H_2_O_2_ mixture and stirred for 1 hour. The resulting suspension was washed with anhydrous ethanol and repeated 3 or 4 times. Following filtration, the obtained precipitate was subjected to vacuum drying for a minimum of 12 hours, yielding the catalyst (P_2_W_18_-DDC).

1 mmol H_3_PW_12_O_40_·*x*H_2_O was dissolved in the 20 mL H_2_O_2_ (30wt%), and solution of 3 mmol of the surfactant DDC in 20 mL of anhydrous ethanol was also prepared. The anhydrous ethanol–DDC mixture was then added to the H_3_PW_12_O_40_–H_2_O_2_ mixture and stirred for 1 hour. The resulting suspension was washed with anhydrous ethanol and repeated 3 or 4 times. Following filtration, the obtained precipitate was subjected to vacuum drying for a minimum of 12 hours, yielding the catalyst (PW_12_-DDC).

### 2.5 Catalyst testing

#### 2.5.1. Catalytic reaction

The selective oxidation of 1-dodecene was carried out in a four-neck round flask (250 mL) equipped with a mechanical agitator which provided a 250 rpm stirring rate and a reflux condenser.

0.1 mol 1-dodecene, 0.1 mol H_2_O_2_ (30% (mass) solution in water), and 5wt% catalysts were added into the flask, the mixture was heated by the heating jacket and stirred by a polytetrafluoroethylene stirring paddle, besides, a mercurial thermometer was immersed into the solution to make accurate temperature measurements. Due to the different reaction conditions of these 2 catalysts, the specific reaction conditions would be provided in results and discussion.

#### 2.5.2. Gas chromatographic and calculation method

The concentration of α-olefin and epoxyalkane were analyzed by gas chromatography (GC-8860A, Angilent, the US) with a flame-ionization detector and a HP-5 column (35 m × 0.32 mm, film: 0.5 mm). The temperature of the injector and detector was 300 °C, and 300 °C respectively. The initial temperature of column was 100 °C for 0 minutes, then raised column temperature at 10 °C per minute to 200 °C for 0 minutes, and 50 °C per minute to 320 °C for 5 minutes at the last. High-purity nitrogen was used as the carrier gas.

Analysis of reaction products were used by external standard method. The conversion of α-olefin and selectivity of epoxyalkane were calculated according to the formula below: Conversion *X* = (*C*_0_ − *C*_*t*_)/*C*_0_, *C*_0_, *C*_*t*_ represents the initial concentration of the reactant and the concentration at time *t* respectively. Yield *γ* = *C*_p*t*_/*C*_p0_, *C*_p*t*_, *C*_p0_ represent the concentration at time *t* and the theoretical concentration of the product respectively. Selectivity *S* = *γ*/*X*. The curves of standard samples were given in ESI (Fig. S1†).

#### 2.5.3. Catalyst characterization

FT-IR analysis: Fourier transform infrared spectroscopy (FT-IR) was used on a VERTEX 70 spectrophotometer (Bruker, GRE) under a dry air condition at room temperature by the KBr pellets method. Scanning electron microscope (SEM) test was performed on TESCAN MIRA LMS. Elemental analysis was performed on Vario EL CUBE. ICP-MS test was performed on Agilent 5110(OES). X-ray photoelectron spectroscopy (XPS) was performed on Thermo Scientific K-Alpha, and the spectra were charge-corrected using a signal located at 284.5 eV. Small-angle X-ray scattering (SAXS) was performed on SAXSess mc2. UV-vis spectra were performed on Hitachi U4150. Powder XRD was performed on D8 ADVANCE A25. Raman spectra analysis were performed on Horiba LabRAM HR Evolution.

## 3. Results and discussion

### 3.1 Catalytic testing

#### 3.1.1. Catalytic performance

Due to the solubility of PW_12_-DDC under high temperature conditions without solvents, the catalytic performance of P_2_W_18_-DDC and PW_12_-DDC in the epoxidation of 1-dodecene was evaluated under the following conditions: 343 K, 1 × 10^−4^ mol catalyst dosage, mechanical stirring at 250 rpm, and a 2 : 1 ratio of H_2_O_2_ to 1-dodecene, dosage of substrate: 0.1 mol. As depicted in Table 1, after 6 hours reaction, P_2_W_18_-DDC exhibited higher selectivity towards dodecane epoxide but lower conversion of 1-dodecene compared to PW_12_-DDC. In contrast to PW_12_-DDC, almost all of the P_2_W_18_-DDC catalyst could be recovered after the reaction.

**Table 1:** The results of direct epoxidation of dodecene catalyzed by different catalyst

No.	Catalyst	Conversion/%	Selectivity/%	Catalyst state
1	PW_12_-DDC	**49**	85	Partial soluble
2	P_2_W_18_-DDC	35	**90**	Insoluble

Subsequently, the catalytic performance of different olefins under identical conditions was investigated (Table S1†). P_2_W_18_-DDC exhibited higher selectivity towards epoxides and lower conversion to olefins, especially notable in the case of 1-tetradecene, which showed 93% selectivity to tetradecane epoxide and 36% conversion. In comparison with other catalysts,^37,38^ P_2_W_18_-DDC demonstrated superior selectivity in the epoxidation of long-chain olefins within a relatively short reaction time (only 6 hours), although with the lower olefin conversion. Additionally, a notable advantage of this catalyst was its ability to maintain high catalytic activity under solvent-free conditions and be readily reused through a straightforward filtration process by anhydrous ethanol, thus reducing production costs and aligning with the concept of green chemistry.

#### 3.1.2. Optimization of reaction conditions

P_2_W_18_-DDC exhibited negligible catalytic activity at temperatures below 323 K. The addition of Wells–Dawson type phosphotungstic acid or surfactants individually into the system did not result in any catalytic activity.

**Fig. 1:**
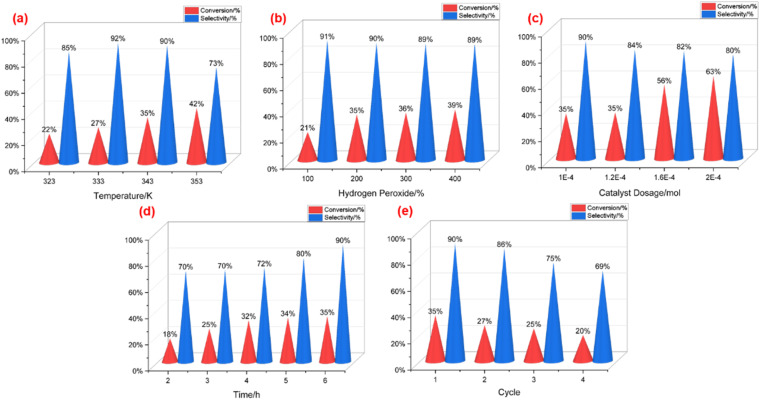
(a) Effect of temperature. Catalyst dosage, 1 × 10^−4^ mol; 250 rpm; ratio of H_2_O_2_ and dodecane: 2 : 1, reaction time: 6 h; (b) effect of initial ratio of H_2_O_2_ and dodecane. Catalyst dosage, 1 × 10^−4^ mol; temperature, 343 K; 250 rpm, reaction time: 6 h; (c) effect of catalyst dosage. Temperature, 343 K; 250 rpm; ratio of H_2_O_2_ and dodecane: 2 : 1, reaction time: 6 h; (d) effect of reaction times. Catalyst dosage, 1 × 10^−4^ mol; temperature, 343 K; 250 rpm; ratio of H_2_O_2_ and dodecane: 2 : 1; (e) reuse of the catalyst. Catalyst dosage, 1 × 10^−4^ mol; 250 rpm; temperature, 343 K; ratio of H_2_O_2_ and dodecane: 2 : 1, reaction time: 6 h.

Initially, the impact of temperature was examined under other invariant reaction conditions (Fig. 1a), with all reaction times kept six hours. As the temperature surpassed 323 K, the conversion of 1-dodecene exhibited a steady increase, while the selectivity of dodecane epoxide initially rose before subsequently declining. At 333 K, P_2_W_18_-DDC demonstrated an optimal selectivity of 92% towards dodecane epoxide. Moreover, the yield of epoxy dodecane continued to rise as the temperature elevated from 323 K to 353 K. Considering both selectivity and product yield, 343 K was selected as the most suitable temperature for the reaction.

Subsequently, the impact of hydrogen peroxide was examined under 343 K with other invariant reaction conditions. The initial ratio of H_2_O_2_ to 1-dodecene was varied from 1 : 1 to 4 : 1. As depicted in Fig. 1b, elevated volumes of hydrogen peroxide led to greater conversion of 1-dodecene but lower selectivity of dodecane epoxide. Notably, the selectivity of dodecane epoxide dropped below 90% when the ratio of hydrogen peroxide to 1-dodecene was 2 : 1. Consequently, an initial ratio of 2 : 1 for H_2_O_2_ and 1-dodecene was deemed the relatively suitable condition.

Next, the influence of catalyst dosage was investigated using four different dosage levels, while keeping other conditions constant. As illustrated in Fig. 1c, P_2_W_18_-DDC exhibited the lowest conversion of 1-dodecene, along with the highest selectivity towards epoxy dodecane, at 1 × 10^−4^ mol catalyst dosage. With an increase in catalyst dosage, the interfacial tension between oil and water decreased due to the presence of long-chain quaternary ammonium salt. This facilitated the formation of smaller droplets and increased the interface area between water phase and oil phase, thereby enhancing the reaction rate. The conversion of 1-dodecene continued to rise, and the selectivity of epoxy dodecane decreased from 90% to 80% as the catalyst dosage increased from 1 × 10^−4^ mol to 2 × 10^−4^ mol. This trend indicated that an elevated amount of catalyst also resulted in the formation of by-products.

Under the determined appropriate conditions, the influence of reaction time was investigated for 2 to 6 hours. As depicted in Fig. 1d, the selectivity of epoxy dodecane exhibited a rapid increase as the reaction progressed, reaching its maximum at 6 hours, while the conversion of 1-dodecene increased at a slower rate.

Finally, cycle experiments were conducted under the specified conditions: Catalyst dosage at 1 × 10^−4^ mol; rotational speed at 250 rpm; temperature at 343 K; and an initial ratio of H_2_O_2_ to 1-dodecene at 2 : 1. The catalyst was subsequently filtered for recycling and washed with anhydrous ethanol at least three times after each reaction cycle. The outcomes were presented in Fig. 1e. As the P_2_W_18_-DDC catalyst was reused, the conversion of 1-dodecene experienced a gradual reduction, while the selectivity of epoxy dodecane dropped steeply after three cycles of recycling. These findings suggested that the catalyst possesses a limited recycling capacity.

### 3.2 Catalyst characterization

#### 3.2.1. Morphology analysis

**Fig. 2:**
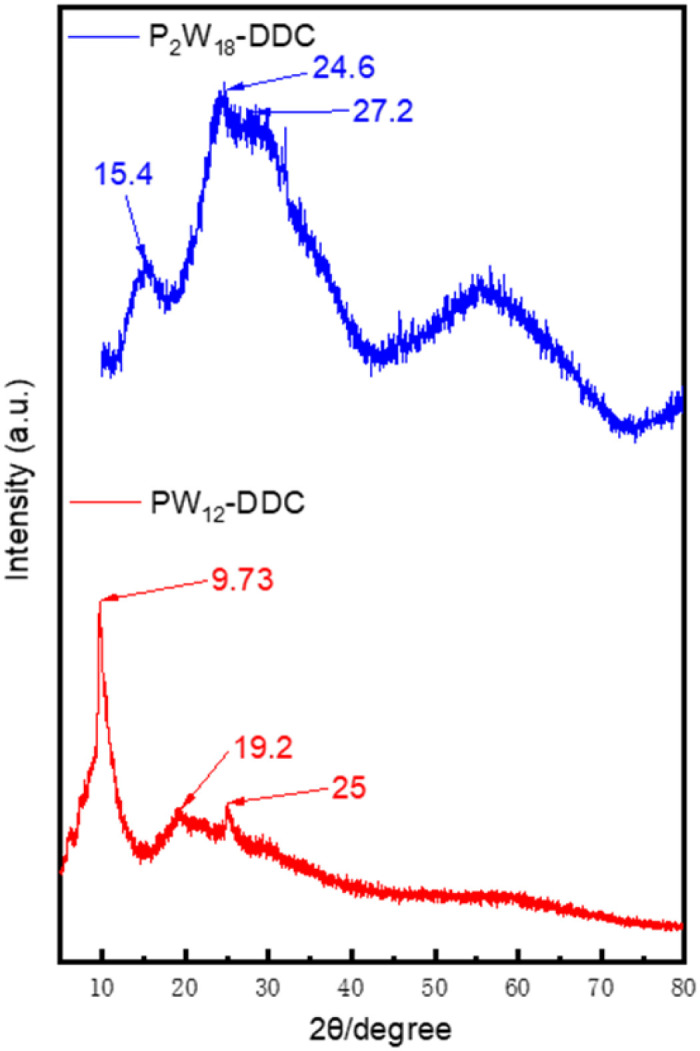
XRD pattern of P_2_W_18_-DDC and PW_12_-DDC.

Small powder XRD was used to reveal the crystal structure of the catalyst. In Fig. 2, both catalysts exhibited amorphous structures, in the XRD pattern of PW_12_-DDC, three different peaks were observed, which were 2 theta of 9.73, 19.2 and 25°, indicated the (110), (220), (222) crystal faces of H_3_PW_12_O_40_ (JCPDS: #50-0304). Compared with H_3_PW_12_O_40_ at 10.62, 20 and 26.7°, the addition of quaternary ammonium salt made the interplanar spacing became larger. P_2_W_18_-DDC displayed only three broad peaks at 15.4, 24.6 and 27.2°, which were consistent with the structure of H_6_P_2_W_18_O_62_.^36^ Compared with P_2_W_18_-DDC, the crystal face of (110) in PW_12_-DDC still performed a strong diffraction, which could be attribute to the polarization of hydrogen bonding between quaternary ammonium salts and phosphotungstic acid. No obvious diffraction peaks of peroxy structure could be observed, which might because that the crystal faces were covered by alkyl chains.^39,40^

**Fig. 3:**
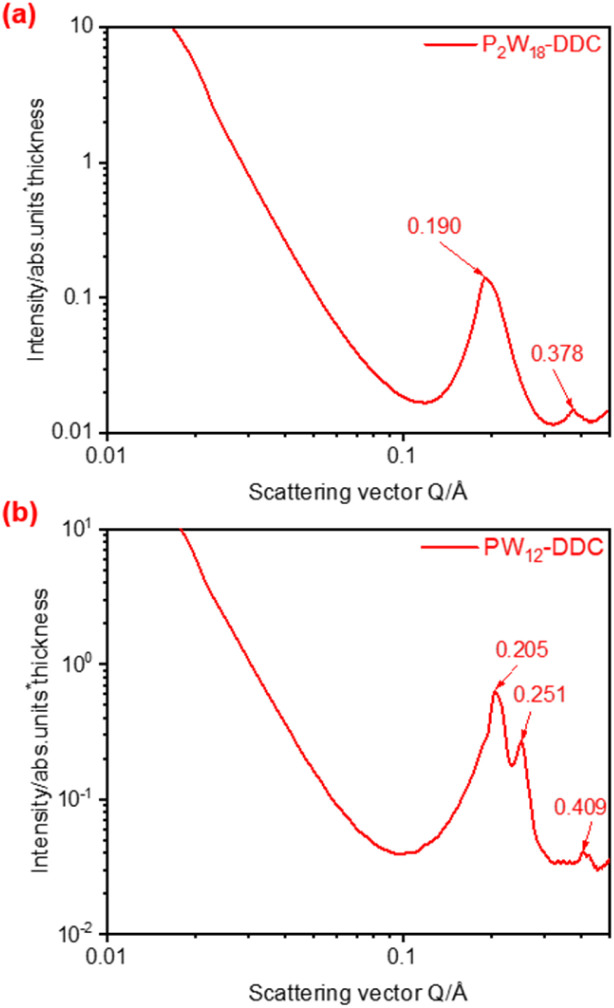
(a) The SAXS pattern of P_2_W_18_-DDC, (b) The SAXS pattern of PW_12_-DDC.

To further determine the microstructure of the catalyst, SAXS analysis was used to characterize P_2_W_18_-DDC and PW_12_-DDC. In the SAXS pattern presented in Fig. 3a, P_2_W_18_-DDC exhibited two scattering peaks arising from ordered structures, characterized by *q* values of 0.190 nm^−1^ and 0.378 nm^−1^, respectively. The nearly 1 : 2 ratio of the q values was indicative of lamellar phases. Conversely, in the SAXS pattern of PW_12_-DDC (Fig. 3b), the *q*-value ratio was approximately 1 : √1.5 : √4, indicating an irregular layered arrangement. The interlayer spacing (*d*-spacing) was determined from the *q*_1_ value of the first scattering peak using the formula *d*_1_ = 2π/*q*_1_. For P_2_W_18_-DDC, the calculated *d* value was 3.3 nm, while for PW_12_-DDC it was 3.06 nm. This observation suggested that P_2_W_18_-DDC possessed a greater layer spacing, which could be attributed to the larger peroxy structure of Wells–Dawson-type phosphotungstic acid. Furthermore, compared with the previously prepared catalys,^35^ quaternary ammonium salts with shorter chain lengths in PW_12_-DDC had performed longer interlayer spacing and more irregular lamellar phases, indicated that with the participation of hydrogen bonding, C–OH in DDC could combine a certain quantity of peroxy phosphotungstic acid, facilitated the binding of a certain quantity of peroxy phosphotungstic acid, resulting in the irregular arrangement between phosphotungstic acid molecules to form PW_12_-DDC, while the larger peroxy structure of Wells–Dawson-type phosphotungstic acid could not accommodate more phosphotungstic acid unit, made the hydrogen bonding ineffective. Due to the enhanced capacity of Wells–Dawson-type phosphotungstic acid to bind quaternary ammonium cations, scanning electron microscopy (SEM) was used to provide detailed micromorphological information. The micrographs of P_2_W_18_-DDC and PW_12_-DDC were presented in Fig. S3 and S4,† respectively. The layered structures of both catalysts were observed, P_2_W_18_-DDC demonstrated a more obvious and denser layered arrangement in comparison to PW_12_-DDC.

#### 3.2.2. Elemental analysis

In theory, 1 mol Wells–Dawson type phosphotungstic acid could theoretically combine with 3 mol surfactant, 1 mol Keggin type phosphotungstic acid could theoretically combine with 1.5 mol surfactant (Table S2†). To determine the relative content of quaternary ammonium salt and phosphotungstic acid of the catalyst, elemental analysis was performed. The elements of C, N, O, and H were conducted using an elemental analyzer, while analysis of P and W was carried out using ICP-MS. The results were presented in Table 2. The molar ratio of W to O in P_2_W_18_-DDC was approximately 2 : 7, and the molar ratio of P to O was nearly 2 : 39. The molar ratio of P to W was 2 : 11. Moreover, the molar ratio of P to N was nearly 4 : 9, indicating that 1 mol Wells–Dawson type phosphotungstic acid molecule was capable of combining with a maximum of 2.25 mol surfactant DDC molecules. This suggested that the charges carried by Dawson type peroxy phosphotungstic acid was lower than the theoretical calculations. Furthermore, in theory, the molar ratio of W to P in PW_12_-DDC was 4 : 1, N to P was nearly 3 : 1, but in fact W to P in PW_12_-DDC was approximately 10 : 1, N to P was nearly 8 : 1, far greater than the ideal structure reported previously.^37^ Although it is reported that the peroxy phosphotungstic acid anion would be converted to a more stable structure of PW_11_/PW_12_ during the immobilization process, more quaternary ammonium salts combined with Keggin type peroxy phosphotungstic acid in PW_12_-DDC might be attributed to the hydrogen bonding between peroxy phosphotungstic acid and surfactant DDC.

**Table 2:** The results of elemental analysis (mol%)

	C/%	H/%	O/%	N/%	P/%	W/%
P_2_W_18_-DDC	24.11	55.67	**14.06**	1.56	**0.68**	**3.92**
PW_12_-DDC	**25.88**	**58.94**	11.45	**1.59**	0.19	1.95

#### 3.2.3. FT-IR analysis

**Fig. 4:**
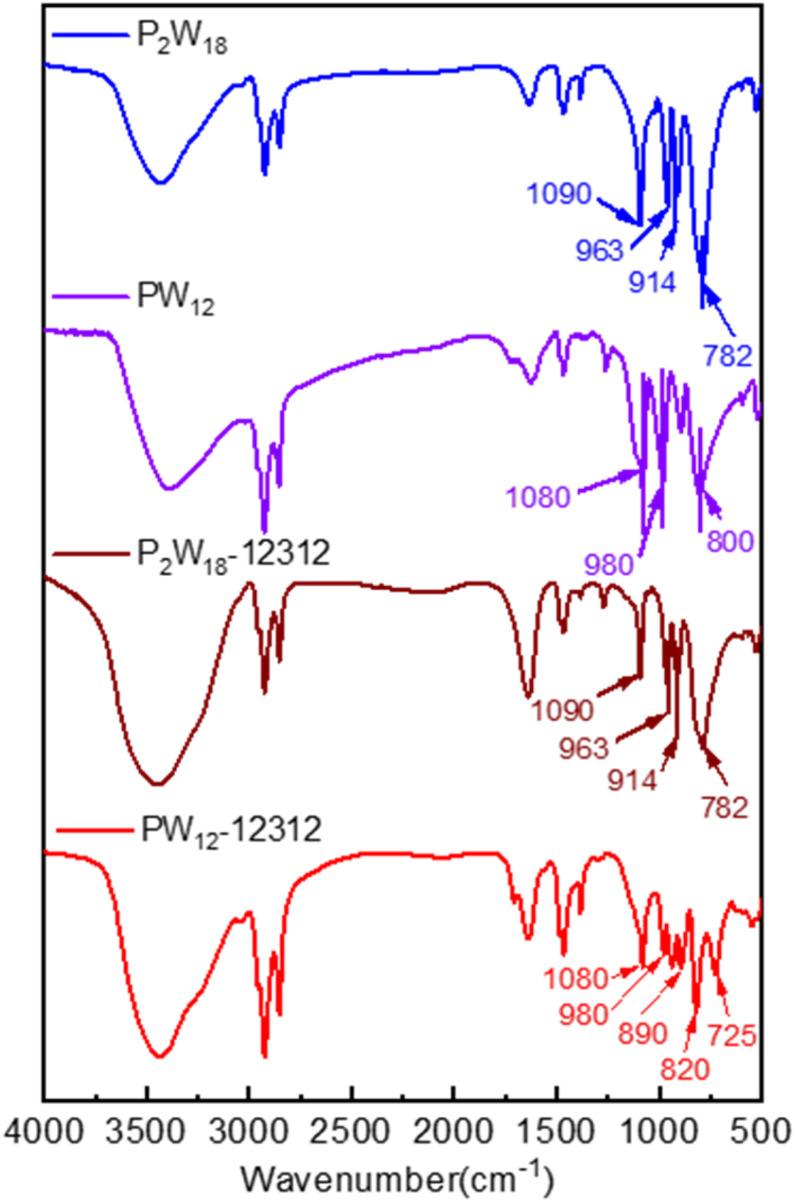
FT-IR spectra of the H_6_P_2_W_18_O_62_, H_3_O_40_PW_12_, P_2_W_18_-DDC and PW_12_-DDC.

FT-IR spectra were used to perform the chemical bonds in the catalyst. In the spectra presented in Fig. 4, both PW_12_-DDC and P_2_W_18_-DDC exhibited peaks at 1470 cm^−1^ (C–N vibration), confirming the successful combination of the surfactant DDC with phosphotungstic acid. The PW_12_-DDC showed the characteristic peak at 1080 cm^−1^ (P–O), 980 cm^−1^ (W=O), 890 cm^−1^ (W–O_b_–W), and 725 cm^−1^ (W–O_c_–W), compared with the Keggin-type phosphotungstic acid at 1080 cm^−1^ (P–O), 980 cm^−1^ (W=O), 890 cm^−1^ (W–O_b_–W), and 800 cm^−1^ (W–O_c_–W), the peaks at 820 cm^−1^ was attributed to the –O–O–, while the peaks of W–O_c_–W performed a red shift from 800 cm^−1^ to 725 cm^−1^, which could not be observed at hydroxy-free quaternary ammonium salt and phosphotungstate acid system, leading to the formation of hydrogen bonding between C–OH and W–O_c_–W, moreover, the new band at 938 cm^−1^ originally branched from 980 cm^−1^ for W=O was assignable to the reduced W^5+^ species due to the intramolecular charge transfer from quaternary ammonium salt to terminal oxygen of PW.^41^ Conversely, the peaks at 914, 963, and 1090 cm^−1^ showed the successful synthesis of Wells–Dawson type of phosphotungstic acid, with the similar peaks in catalyst P_2_W_18_-DDC, they exhibited common corresponding to the vibration of W–O_b_–W, W=O, and P–O, respectively. In conjunction with the elemental analysis results, treatment with H_2_O_2_ resulted in the conversion of partial H_6_P_2_W_18_O_62_ into a peroxy structure. No significant shift was observed in P_2_W_18_-DDC, indicating that the combination of Dawson type phosphotungstic acid and quaternary ammonium salts occurred solely through ionic bonding. Furthermore, both catalysts retained the characteristic peak of W–O_c_–W, confirming the preservation of their Keggin and Wells–Dawson type phosphotungstic acid structures.

#### 3.2.4. XPS analysis

**Fig. 5:**
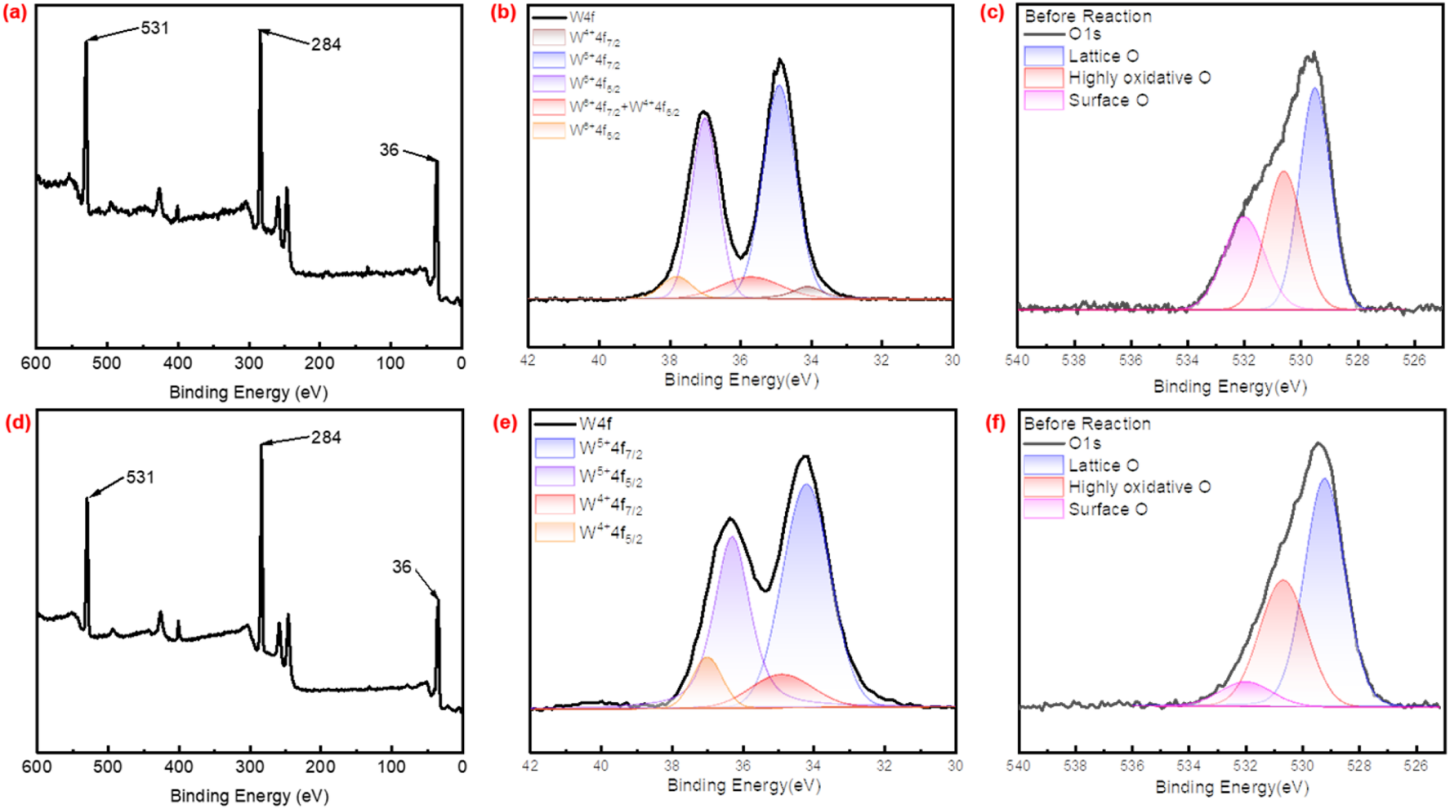
(a) The full XPS spectra of the P_2_W_18_-DDC, (b) the W 4f XPS spectra of P_2_W_18_-DDC, (c) the O 1s XPS spectra of P_2_W_18_-DDC (d) the full XPS spectra of the PW_12_-DDC, (e) the W 4f XPS spectra of PW_12_-DDC, (f) the O 1s XPS spectra of PW_12_-DDC.

The XPS spectra were used to reveal the elemental composition on the surface of catalysts. In the XPS spectra shown in Fig. 5a, the full spectra of P_2_W_18_-DDC exhibited obvious peaks at 531, 284, and 36 eV, corresponding to the O 1s, C 1s, and W 4f signals respectively. Similar peaks were observed for PW_12_-DDC as shown in Fig. 5. Following the peaks separation method described by Zhao, *et al.*^42^Fig. 5b revealed the fitting of the W element in the catalyst into five distinct W 4f peaks. Specifically, the peak at 34 eV for W 4f_5/2_ were attributed to W^4+^, the peak at 34.9 eV for W 4f_7/2_ and 37 eV for W 4f_5/2_ were attributed to W^5+^, while the peaks at 35.8 eV for W 4f_7/2_ and 37.9 eV for W 4f_5/2_ were attributed to W^6+^. The surface elemental content of W^5+^ in P_2_W_18_-DDC was approximately 79.71%, with the elemental content of W^4+^ was 8.18%. In comparison with Wells–Dawson type phosphotungstic acid, a portion of W on the surface of P_2_W_18_-DDC was reduced to W^5+^, suggesting the disruption of the Wells–Dawson type phosphotungstic acid structure on the surface, which could combine with more negative charges, increase the interaction between surfactant and WO_*X*_. But the W 4f signal in PW_12_-DDC in Fig. 5e was divided into 4 peaks, the peak at 34 eV for W 4f_5/2_ and the peak 36.1 eV for W 4f_7/2_ were attributed to W^4+^, the peak at 34.9 eV for W 4f_5/2_and the peak at 37 eV for W 4f_7/2_ were attributed to W^5+^, while the W^6+^ species were not observed.

In the O 1s spectra in Fig. 5c and f, the peak fitting revealed three distinct peaks, the peak at 529.5 eV was attributed to lattice oxygen, while the peak at 530.5 eV corresponded to highly oxidative oxygen species, the peak at 531.5 eV was attributed to surface oxygen species. The surface O in catalyst could be classified as reaction intermediates and other active O species, such as W(O)_2_ and less C–OH of quaternary ammonium salt, while W=O could be attributed to highly oxidative O, which was reported to be closely correlated to surface O, could participate in the reaction by combining O to become an active substance, W–O–W could be attributed to lattice O.^43,44^ During the catalytic reaction, only surface oxygen, along with highly oxidative oxygen participate in the reaction process. It was worthy noting that although the relative content of quaternary ammonium salts in PW_12_-DDC was relatively higher than P_2_W_18_-DDC, the content of surface O specie was just 9.36%, lower than 24.87% in P_2_W_18_-DDC, but the content of highly oxidative O specie and FWHM were higher than P_2_W_18_-DDC, which could be attributed to the formation of hydrogen bonding between phosphotungstic acid and surfactant DDC during the preparation of PW_12_-DDC.

### 3.3 Mechanism analysis

#### 3.3.1. SEM analysis after reaction

The utilized P_2_W_18_-DDC was further categorized into two groups: unwashed (Fig. S5†) and washed with anhydrous ethanol (Fig. S5†). Both exhibited distinct lamellar phases, underscoring the catalysts' insolubility in both oil and water phases. In Fig. S5,† it was evident that the majority of olefins and epoxides were deposited on the catalyst surface, while the Fig. S6† illustrated that a significant portion of 1-dodecene was effectively removed by anhydrous ethanol washing while maintaining the layered accumulation. The SEM picture of utilized PW_12_-DDC was shown in Fig. S7,† although the layered structure could still be observed on the catalyst, the layered phase structure performed less distinct, which could be attributed to the further coverage of quaternary ammonium salts and the loss of phosphotungstic acid. To confirm this conclusion, relevant characterizations were analyzed in the following sections.

#### 3.3.2. FT-IR analysis after reaction

**Fig. 6:**
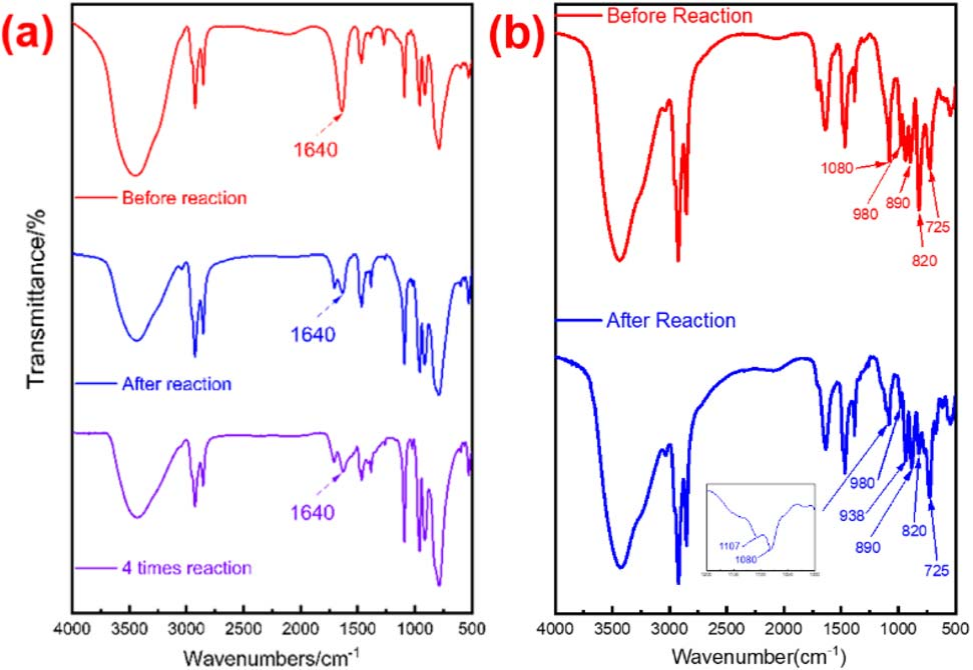
(a) FT-IR spectra of P_2_W_18_-DDC, (b) FT-IR spectra of PW_12_-DDC.

In the FT-IR spectra of the utilized P_2_W_18_-DDC (Fig. 6), the structural features remained largely unaltered after the reaction. The characteristic peaks at 914, 963, and 1090 cm^−1^, attributed to the vibrations of W–O_b_–W, W=O, and P–O, respectively, were still prominently present. Interestingly, in comparison to after reaction P_2_W_18_-DDC, the majority of the W=O peaks and –O–O– peaks in PW_12_-DDC nearly vanished (at 980 cm^−1^ and 820 cm^−1^), leaving only very faint residual peaks, the peak of W–O_c_–W at 725 cm^−1^ increased noticeably, which was not observed before.^35^ The reason for these significant conversion among W=O, W(O_2_) and W–O_c_–W might not only be due to the transformation from W=O to W(O_2_), the peroxidation from W–O_c_–W to W(O_2_) induced by hydrogen bonding also involved in the reaction during the catalytic reaction. Furthermore, the peaks at 1080 cm^−1^ and 1107 cm^−1^ also confirmed the formation of hydrogen bonding among the terminal oxygen atoms of PW_12_ anions and the quaternary ammonium salt,^45^ leading to the redox properties of W species tuned by W–O⋯H.

#### 3.3.3. XPS analysis after reaction

In the XPS spectra of C 1s in P_2_W_18_-DDC and PW_12_-DDC before and after reaction (Fig. S8†), the C–N content of these two catalysts remained basically unchanged, proving that the use of anhydrous ethanol could effectively wash off residual olefins and epoxides from the catalyst surface.

The XPS spectra of O 1s and W 4f of utilized P_2_W_18_-DDC and PW_12_-DDC were demonstrated in Fig. S9.† After reaction, the ratio of surface O (C–OH and W(O_2_)) to lattice O (W–O–W) for the catalyst was lower than before the reaction in both catalysts. This reduction in ratio likely contributed to the decrease in catalytic reactivity of catalysts, but the content of highly oxidative O nearly unchanged, indicating the transformation between W(O_2_) and W=O. Notably, after the reaction, the content of W^6+^ in P_2_W_18_-DDC have been increased with the decreased content of W^4+^(Fig. S9d†), with a narrower FWHM of W^5+^ peaks in 34.9 eV. This observation suggested that during the reaction, some of the W^5+^ and W^4+^ species were oxidized back to higher valence states. Conversely, in the W 4f spectra of PW_12_-DDC (Fig. S9b†), the surface tungsten (W) species of PW_12_-DDC predominantly existed in the +5 valence state or lower. After the reaction, a decrease in W^5+^ contents have been observed, while the content of highly oxidative O and lattice O increased obviously, indicating the formation of WO_*X*_ groups instead of the original structure of phosphotungstic acid,^44^ leading to the weakening of layered structure observed on the SEM image.

#### 3.3.4. UV-vis analysis after reaction

The UV-visible spectra of P_2_W_18_-DDC are presented in Fig. S10.† Notably, a region of higher energy, ranging from 210 to 230 nm, was observed in Fig. S10a,† which could be attributed to charge transfer between the metal and oxygen species, as also indicated at 238 nm in Fig. S10b.†^45^ After the reaction of P_2_W_18_-DDC (Fig. S10a†), it is noteworthy that the peak at 300 nm experienced a blue shift to 290 nm. Additionally, the absorption edge extended from approximately 480 nm to 430 nm, indicating the oxidation of the metal center to a relatively higher valence state, conversely, the absorption edge of PW_12_-DDC experienced a subtle red shift, indicating the transfer to a lower valence state of the metal center, which was consistent with the results of XPS analysis.

#### 3.3.5. Raman spectra analysis

**Fig. 7:**
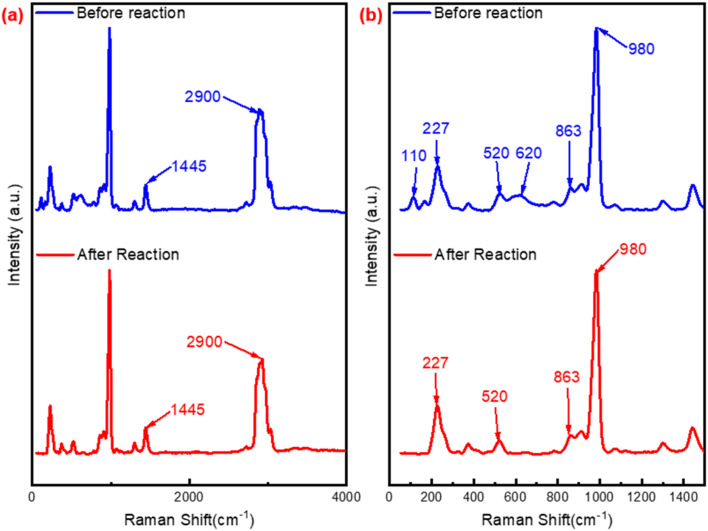
Raman spectra of P_2_W_18_-DDC and used P_2_W_18_-DDC (a) range from 50 to 4000 cm^−1^; (b) range from 50 to 1500 cm^−1^.

**Fig. 8:**
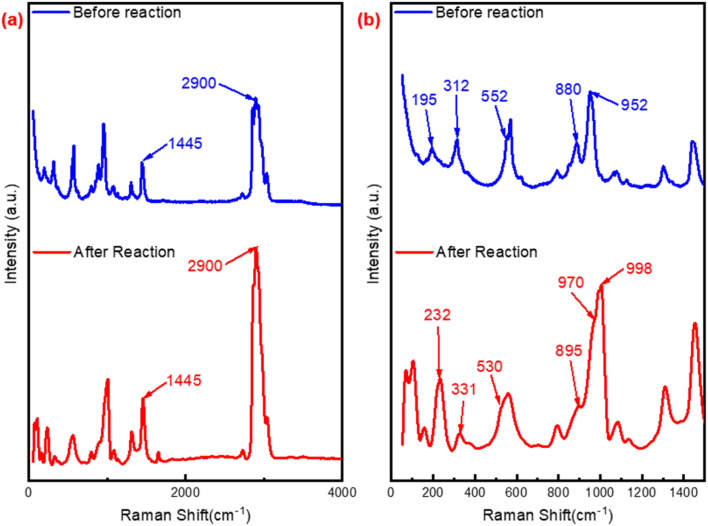
Raman spectra of PW_12_-DDC and used PW_12_-DDC (a) range from 50 to 4000 cm^−1^; (b) range from 50 to 1500 cm^−1^.

The Raman spectra of utilized P_2_W_18_-DDC and PW_12_-DDC were also performed. As depicted in Fig. 7, after reaction, the disappearance of the peaks at 110 cm^−1^ (structure of Wells–Dawson type phosphotungstic acid) and 620 cm^−1^ (vibration of C–O) indicated the formation of more peroxy structure in P_2_W_18_-DDC. However, the PW_12_-DDC showed noticeable change after reaction (Fig. 8). Firstly, compared with Keggin type phosphotungstic acid (Fig. S11†), the tungsten correlation peaks at 195, 312 and 552 cm^−1^ in PW_12_-DDC showed a red shift, which could be contributed the hydrogen bonding between W–O–W and C–OH,^46^ while P_2_W_18_-DDC showed a blue shift, indicating the larger groups of peroxy structure in P_2_W_18_-DDC, making the influence of hydrogen bonding ineffective. It could be observed the peaks at 1445 cm^−1^ and 2900 cm^−1^ intensified, leading to the removal of phosphotungstic acid structure. The peaks at 880 and 952 cm^−1^ have performed a red shift to 895 and 970 cm^−1^, indicating the transformation from W(O)_2_ to W=O,^47^ the blue shift from 552 to 530 cm^−1^ revealed the transform between W–O_c_–W and W(O)_2_, while the red shift from 195 to 232 cm^−1^, 312 to 331 cm^−1^ could also confirm this conversion. Furthermore, compared with P_2_W_18_-DDC, two new peaks at 100 cm^−1^ and 998 cm^−1^ were observed in the PW_12_-DDC, indicated that during the catalytic reaction, the peroxy structure of catalyst underewent a process of forming WO_*X*_ groups influenced by hydrogen bonding between DDC and phosphotungstic acid,^43^ while the P_2_W_18_-DDC tended to form the peroxy structure. Combined with the results of FT-IR, XPS spectra and Raman spectra, after the reaction, a portion of the W–O–W/W=O species involved in the reaction might not return to their original structure, but rather combine with the remaining C–OH in quaternary ammonium salts through hydrogen bonding to form groups like WO_3/4_.

#### 3.3.6. Possible reaction route

**Fig. 9:**
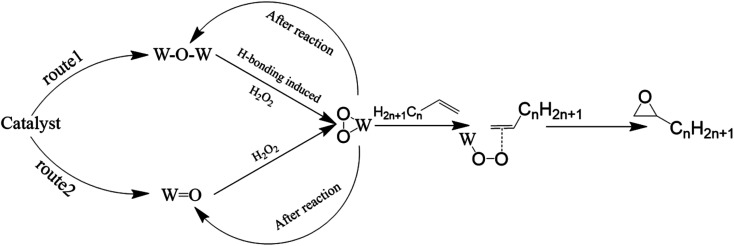
Reaction route of catalysts.

In summary, there exist two distinct catalytic reaction pathways in these catalysts, which was shown in Fig. 9. In the epoxidation reaction catalyzed W–O–W species, due to the hydrogen bonds combined with W–O–W and C–OH, the electrophilicity of W–O–W species were enhanced by hydrogen bonding,^48,49^ a portion of W–O–W could combine with oxygen to form W(O_2_), then those peroxy structure and W(O_2_) formed from W=O (phosphotungstic acid) participates in the catalytic reaction. After the reaction, the W(O_2_) species transform into W–O–W to next catalytic reaction. Conversely, in the epoxidation reaction catalyzed by W=O species, the reaction pathways were the same as previous study,^37^ the W=O species combine with oxygen to form W(O_2_). After the reaction, a portion of the W(O_2_) species revert to W=O, while the remaining W(O_2_) species retain their hypervalent state structure. By using various characterization methods, it is proved that the catalyst prepared by Keggin type phosphotungstic acid would work through the above two routes in the catalytic reaction. While there was only one route (W(O_2_) formed from W=O) worked in catalytic reaction of the catalyst prepared by Wells–Dawson type phosphotungstic acid.

## 4. Conclusions

In this study, two new types of catalyst was prepared to utilize Wells–Dawson-type phosphotungstic acid and Keggin type phosphotungstic acid combined with a biquaternary ammonium cation. Various characterizations were used to elucidate the fundamental structure of the catalysts. Compared with a catalyst synthesized with Keggin-type phosphotungstic acid, the Wells–Dawson-type phosphotungstic acid catalyst exhibited superior selectivity and recyclability, but lower conversion in the epoxidation of 1-dodecene. Subsequently, the impact of various reaction conditions on catalyst performance was investigated, leading to the determination of optimal reaction conditions under solvent-free circumstances, thereby highlighting its potential industrial applicability. Throughout the course of the reaction, significant structural alterations in the catalyst were observed. Consequently, a comparative analysis was conducted between the pre- and after-reaction states, enabling the deduction of the catalytic mechanism influenced by hydrogen bonds for the catalyst synthesized with Keggin type phosphotungstic acid. Notably, this mechanism revealed a distinct reaction pathway in contrast to catalysts synthesized with Wells–Dawson type phosphotungstic acid.

## Conflicts of interest

The authors declare that they have no known competing financial interests or personal relationships that could have appeared to influence the work reported in this paper.

## Supplementary Material

RA-014-D4RA01399A-s001
