# Supplementary material for: The influence of hydrogen bonding on the structure of organic–inorganic hybrid catalysts and its application in the solvent-free epoxidation of α-olefins
Source: RSC Adv. 2024 Apr 22;14(18):12853–63. doi: 10.1039/d4ra01399a (PMC11033607; doi:10.1039/d4ra01399a)
Supplement: RA-014-D4RA01399A-s001 [file RA-014-D4RA01399A-s001.pdf]

Electronic Supplementary Material (ESI) for RSC Advances.
This journal is © The Royal Society of Chemistry 2024

Supporting Information

for

The influence of hydrogen bonding on the structure of organic-inorganic hybrid catalysts and its application in the solvent-free epoxidation of α-olefins

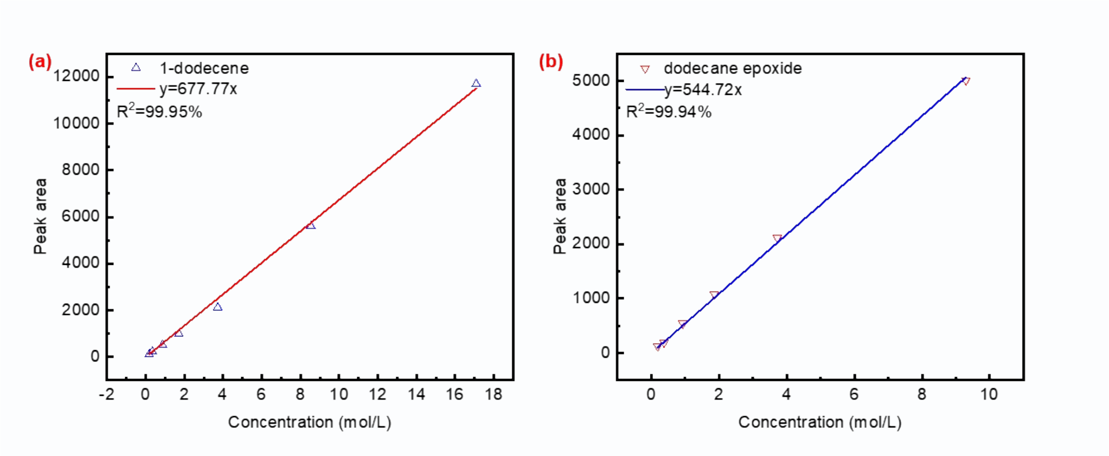

Fig.S1. Curves of standard samples by Gas chromatographic, (a) 1-dodecene, (b) dodecane epoxide

Table S1. Catalytic reaction of different olefins

Substrate	Product	Selectivity/%	Conversion/%
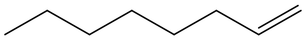	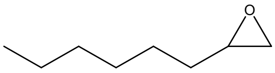	80.8	26.2
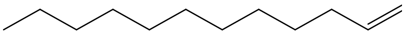	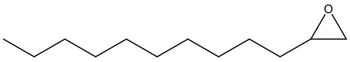	90.5	29.8
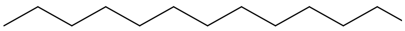	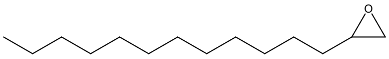	93.4	35.8
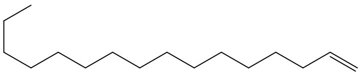	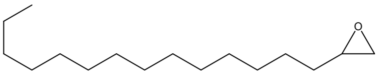	92.1	28

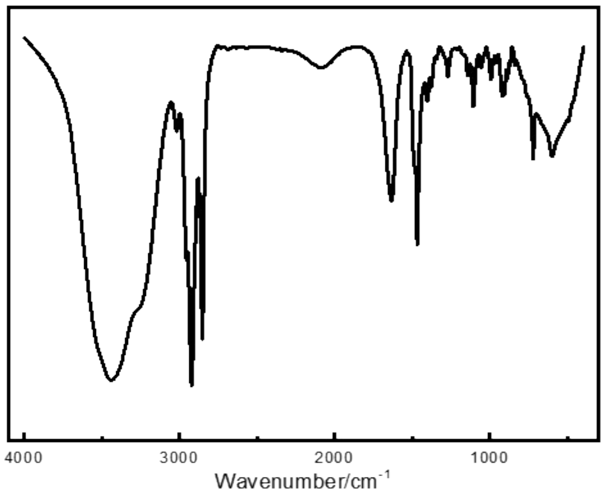

Fig.S2. FT-IR spectra of DDC; 3437 cm^{-1} (O-H stretching vibration), 2921 cm^{-1} (C-H asymmetrical stretching vibration), 2853cm^{-1}(C-H stretching vibration), 1469cm^{-1}(C-N stretching vibration), 1403 cm^{-1} (-CH_2- bending vibration)

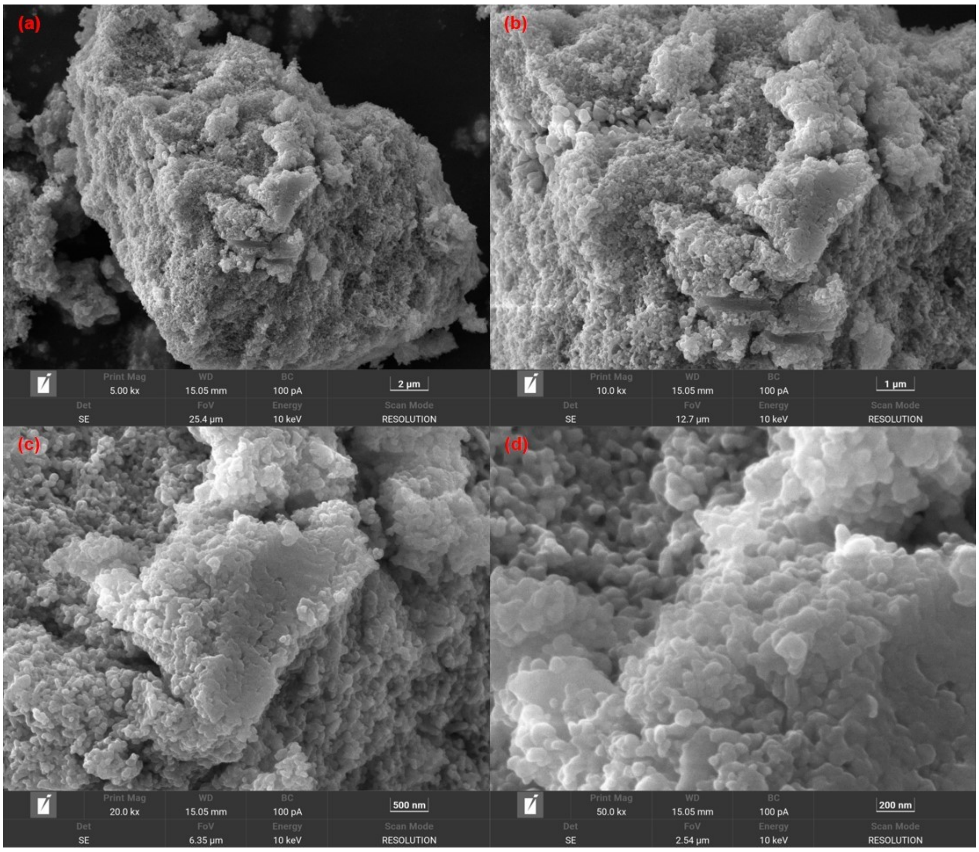

Fig.S3. The SEM of P_2W_{18}-DDC (a) 2μm, (b) 1μm, (c) 500nm, (d) 200nm

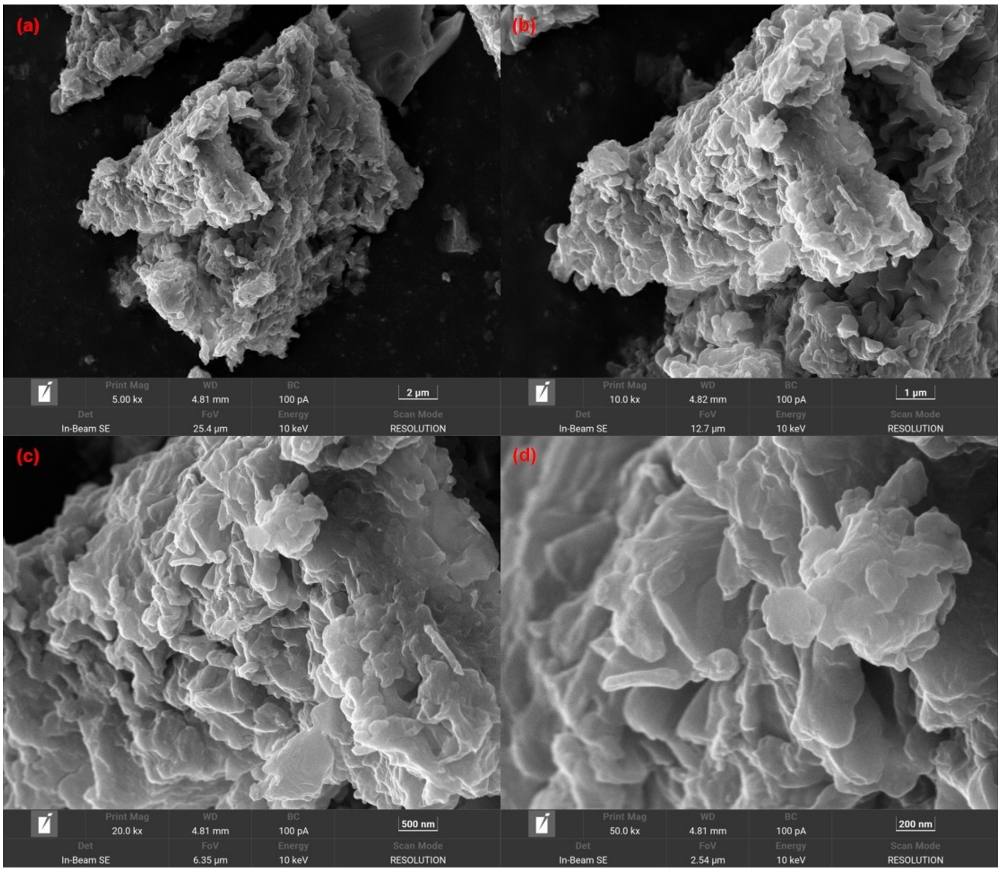

Fig.S4. The SEM of PW_{12}-DDC (a) 2μm, (b) 1μm, (c) 500nm, (d) 200nm

Table.S2. Molar ratio of P:N and P:W in two catalysts

	P:N	P:W
P_2W_{18}-DDC	4:9	2:11
P_2W_{18}-DDC (In theory)	1:3	1:3
PW_{12}-DDC	1:8	1:10
PW_{12}-DDC (In theory)	2:3	1:4

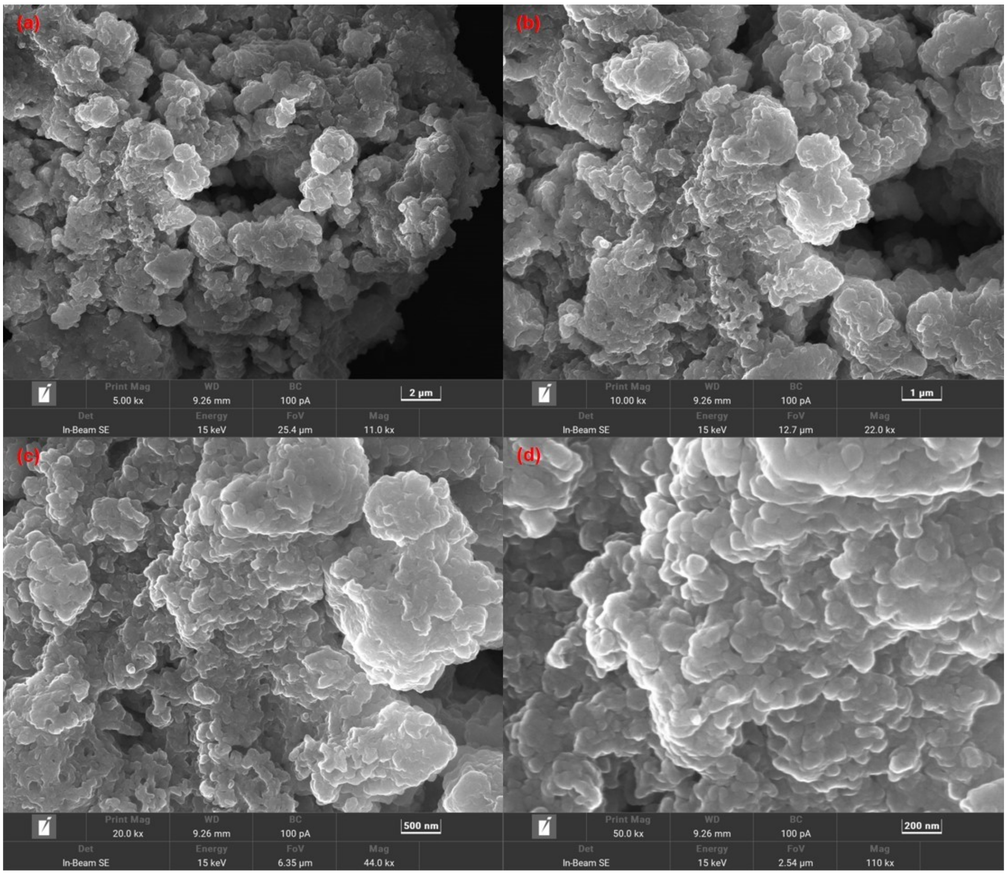

Fig.S5. The SEM of used P_2W_{18}-DDC (unwashed) (a) 2μm, (b) 1μm, (c) 500nm, (d) 200nm

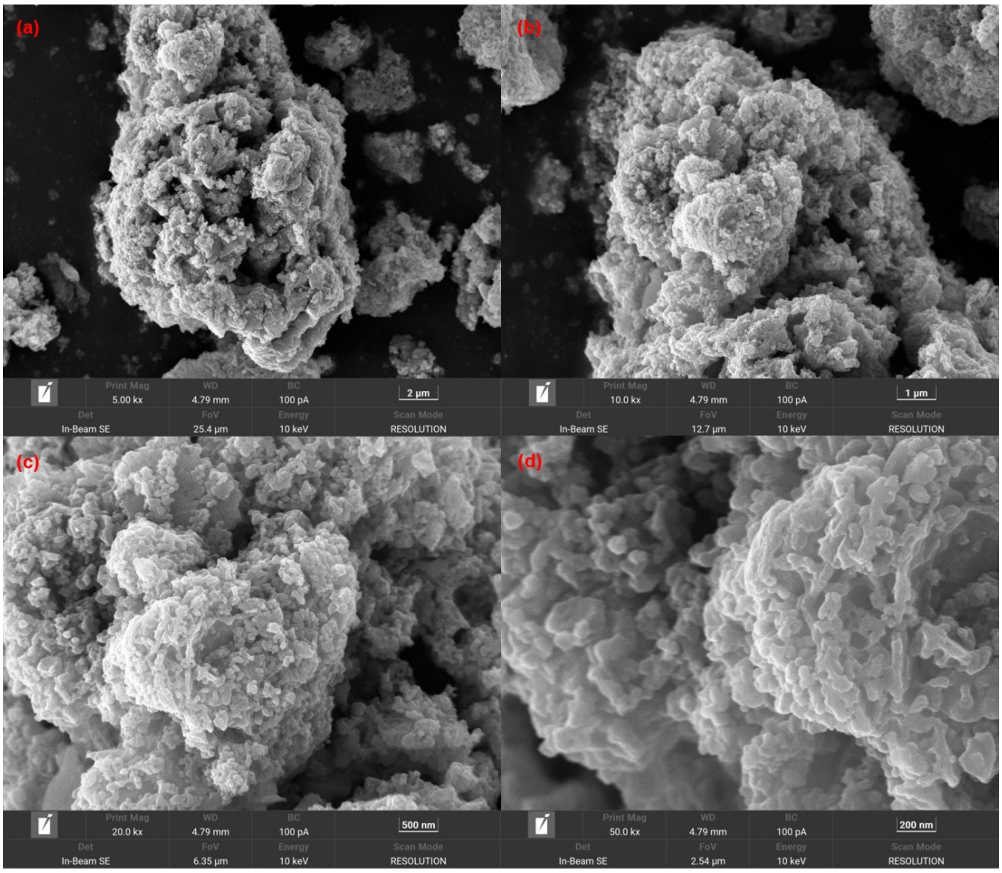

Fig.S6. The SEM of used P_2W_{18}-DDC (washed) (a) 2μm, (b) 1μm, (c) 500nm, (d) 200nm

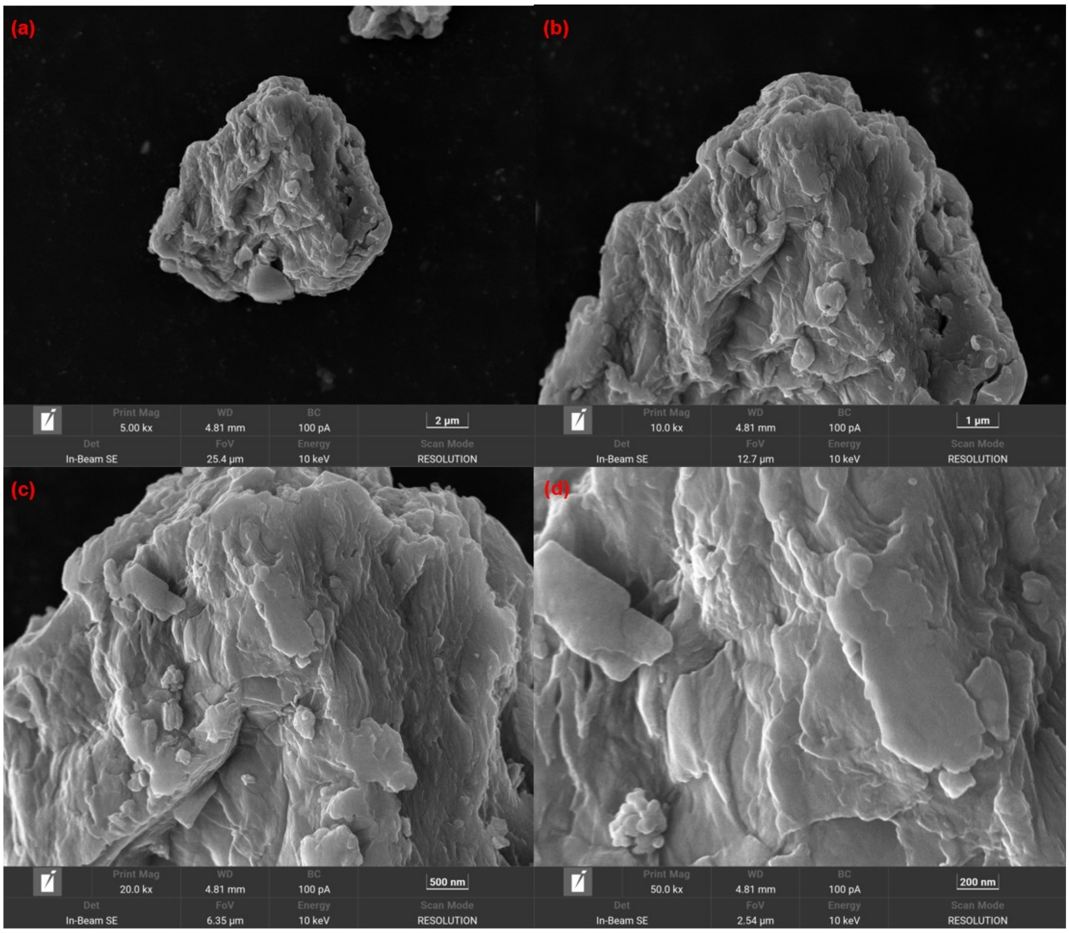

Fig.S7. The SEM of used PW12-DDC (washed) (a) 2μm, (b) 1μm, (c) 500nm, (d) 200nm

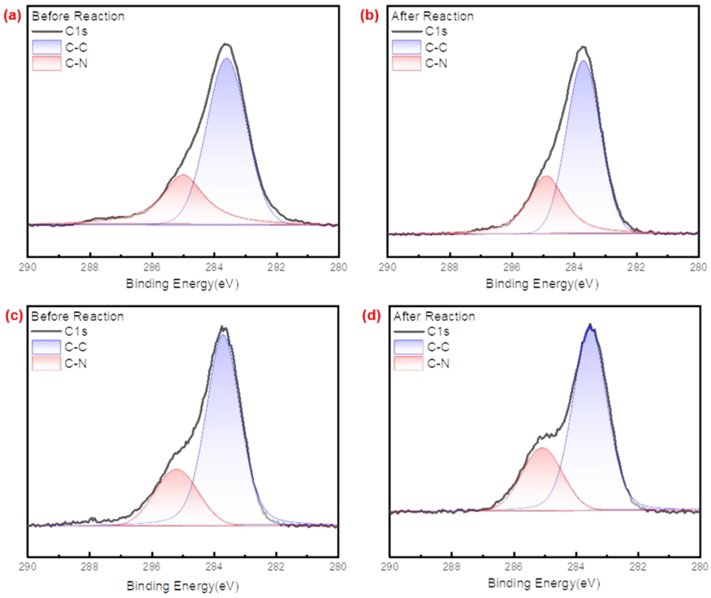

Fig.S8. (a) the C1s XPS spectra of PW_{12}-DDC, (b) the C1s XPS spectra of used PW_{12}-DDC, (c) the C1s XPS spectra of P_2W_{18}-DDC, (d) the C1s XPS spectra of used P_2W_{18}-DDC

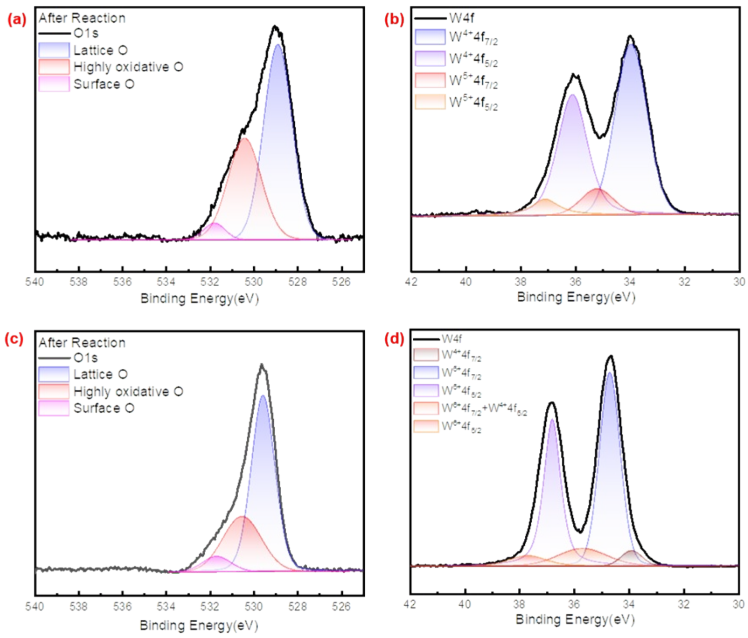

Fig.S9. (a) the O1s XPS spectra of used PW_{12}-DDC, (b) the W4f XPS spectra of used PW_{12}-DDC, (c) the O1s XPS spectra of used P_2W_{18}-DDC, (d) the W4f XPS spectra of used P_2W_{18}-DDC

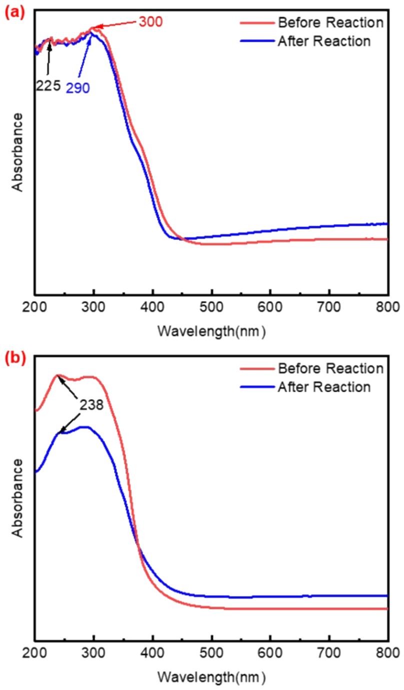

Fig.S10. (a) UV-Vis spectra of P_2W_{18}-DDC, (b) UV-Vis spectra of PW_{12}-DDC

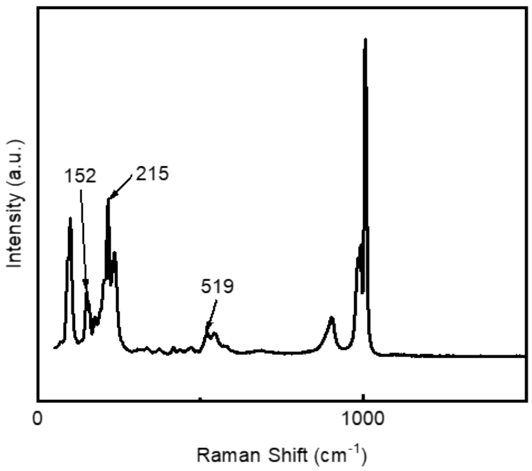

Fig.S11. Raman spectra of $H_3PW_{12}O_{40}$
